# 5-fluorouracil steady state pharmacokinetics and outcome in patients receiving protracted venous infusion for advanced colorectal cancer

**DOI:** 10.1054/bjoc.2000.1664

**Published:** 2001-03

**Authors:** D I Jodrell, M Stewart, R Aird, G Knowles, A Bowman, L Wall, J Cummings, C McLean

**Affiliations:** ICRF Medical Oncology Unit, University of Edinburgh; Directorate of Clinical Oncology and Haematology, Western General Hospital, Edinburgh, UK

**Keywords:** protracted infusion 5FU, 5FU pharmacokinetics, colorectal cancer

## Abstract

PVI 5FU gives increased response rates and reduced toxicity when compared to bolus 5FU (J Clin Oncol 1989, 425–432). PVI 5FU administration was reported to give highly variable (>1000-fold) plasma 5FU concentrations at steady state (FU Css) which correlated with toxicity (Ann Oncol 1996, 47–53); but only 19 patients were studied. Therefore, we performed a study of PVI 5FU in 61 patients with advanced colorectal cancer to assess the variability (inter- and intra-subject) in 5FU Css associated with PVI 5FU (300 mg m^−2^day^−1^) and to attempt to correlate pharmacodynamic end-points (anti-tumour activity, toxicity) with 5FU Css as a prelude to ‘exposure-guided’ 5FU administration. All 5FU sampling was performed between 10 am and noon. PVI 5FU administration continued to 26 weeks in patients with disease improvement or stabilization. The response rate was 26% (33% stable disease) and median survival was 11 months. Hand–foot syndrome was the most common dose limiting toxicity. Variability in 5FU _300_ Css was considerably less than previously reported; 94 ± 25 ng ml^−1^(CV = 27%). No relationships were demonstrated between subject mean 5FU _300_ Css and PD end-points such as response, mucositis, diarrhoea and hand–foot syndrome. The lack of correlation suggests that measurement of 5FU concentrations should not be used to individualize dosing in patients receiving PVI 5FU for advanced colorectal cancer. © 2001 Cancer Research Campaign http://www.bjcancer.com


*British Journal of Cancer* (2001) **84**(5), 600–603
© 2001 Cancer Research Campaign
doi: 10.1054/ bjoc.2000.1664, available online at http://www.idealibrary.com on IDEAL

http://www.bjcancer.com

### 5-fluorouracil steady state pharmacokinetics and outcome in patients receiving protracted venous infusion for advanced colorectal cancer

**DI Jodrell[1], M Stewart[1], R Aird[1], G Knowles[1], A Bowman[2], L Wall[1], J Cummings and C McLean[2]**

[1]ICRF Medical Oncology Unit, University of Edinburgh and [2]Directorate of Clinical Oncology and Haematology, Western General Hospital, Edinburgh, UK

**Summary** PVI 5FU gives increased response rates and reduced toxicity when compared to bolus 5FU (J Clin Oncol 1989, 425–432). PVI 5FU administration was reported to give highly variable (>1000-fold) plasma 5FU concentrations at steady state (FU Css) which correlated with toxicity (Ann Oncol 1996, 47–53); but only 19 patients were studied. Therefore, we performed a study of PVI 5FU in 61 patients with advanced colorectal cancer to assess the variability (inter- and intra-subject) in 5FU Css associated with PVI 5FU (300 mg m$^{-2}$ day$^{-1}$) and to attempt to correlate pharmacodynamic end-points (anti-tumour activity, toxicity) with 5FU Css as a prelude to 'exposure-guided' 5FU administration. All 5FU sampling was performed between 10 am and noon. PVI 5FU administration continued to 26 weeks in patients with disease improvement or stabilization. The response rate was 26% (33% stable disease) and median survival was 11 months. Hand–foot syndrome was the most common dose limiting toxicity. Variability in $5FU_{300}$Css was considerably less than previously reported; 94 ± 25 ng ml$^{-1}$ (CV = 27%). No relationships were demonstrated between subject mean $5FU_{300}$Css and PD end-points such as response, mucositis, diarrhoea and hand–foot syndrome. The lack of correlation suggests that measurement of 5FU concentrations should not be used to individualize dosing in patients receiving PVI 5FU for advanced colorectal cancer. © 2001 Cancer Research Campaign http://www.bjcancer.com

**Keywords**: protracted infusion 5FU; 5FU pharmacokinetics; colorectal cancer

5-fluorouracil (5FU) is the drug most commonly used in the treatment of patients with colorectal cancer. However, 5FU is active only during S-phase of the cell cycle and therefore its activity may be limited by its short plasma half-life in man. Indeed, response rates in patients with advanced colorectal cancer using bolus schedules of 5FU are very poor (10–15%) and attempts have been made to improve this by modification of the schedule of administration. A response rate of 30% has been achieved using a protracted venous infusion (PVI) schedule (Lokich et al, 1988) at a standard dosage (300 mg m$^{-2}$ day$^{-1}$) and this mode of administration is well tolerated in terms of toxicity. A recent meta-analysis of 1219 patients included in 6 randomized trials also supports the use of infusional 5FU compared to bolus 5FU (Meta-analysis Group in Cancer, 1998).

The plasma concentration of 5FU at steady state (5FU Css) during PVI 5FU has been reported as being highly variable (<25–>25 000 ng ml$^{-1}$) and it has also been suggested that this correlates with the incidence of toxicity (Findlay et al, 1996). However, Findlay et al reported data from only 19 patients and therefore the phase II clinical study reported here was designed to further investigate this issue. Relationships between 5FU exposure and outcome have been reported for patients with head and neck cancer. Santini et al reported 2 sequential cohorts of patients in whom dose modification was made based on 5FU exposure (Santini et al, 1989). Santini suggested an increase in therapeutic index as patients appeared to suffer less toxicity and the complete response rate was also improved. However, this was a non-randomized study and differences in the tumour stage of patients in the 2 cohorts, may have explained the differences in outcome. Subsequently, a prospective randomized trial has been performed and in this trial, although survival was no different between the arms, the utility of monitoring 5FU exposure to reduce toxicity was confirmed (Fety et al, 1997).

A relationship between 5FU dose intensity and therapeutic response has been described in patients with colorectal cancer by Gamelin et al (1998). In this study, 5FU dosage was optimised using pharmacokinetic monitoring. This was a phase II trial incorporating 152 patients, and this strategy was associated with a high response rate (43%) and a median survival of 19 months. This trial did not have a standard therapy control arm, but did appear to show some association between 5FU concentration and outcome. An additional factor regulating 5FU pharmacokinetics is circadian variation (Harris et al, 1990). Analysis of 5FU concentrations during continuous infusion therapy, identified 11 am as the time of peak 5FU levels and this was inversely related to leucocyte DPD activity, where trough activity levels were seen at 11 am in the same patients (Harris et al, 1990).

Therefore, the aims of this study were i) to confirm the antitumour activity of PVI 5FU, ii) to document the toxicities associated with PVI 5FU, iii) to assess the variability in 5FU Css within the study population and iv) to correlate pharmacodynamic end-points, such as antitumour activity and toxicity, with the 5FU Css in individual patients.

*Received 7 February 2000*
*Revised 6 October 2000*
*Accepted 5 December 2000*

*Correspondence to:* DI Jodrell

### METHODS

Patients were recruited from the combined Colorectal Cancer Clinic at the Western General Hospital (WGH), Edinburgh, UK.

To be eligible, patients had to have a histologically proven diagnosis of colorectal cancer which had relapsed following primary surgery or was advanced (metastatic disease) at the time of presentation. Patients had to be capable of understanding the nature of the trial and gave written informed consent. The protocol had been reviewed and approved by the Lothian Research Ethics Committee (1702/95/4/20). Patients must have received no prior chemotherapy for metastatic disease and had adequate performance status (CTC grade 0–2). Patients had adequate renal function as assessed by serum creatinine ≤120 μM or calculated creatinine clearance ≥60 ml $min^{-1}$ and liver function tests (bilirubin, transaminases) were within the WGH laboratory normal range, unless associated with metastatic disease. The patients' life expectancy was at least 3 months and all patients were aged ≥18 years. The dose of 5FU was 300 mg $m^{-2}$ $day^{-1}$. This was administered via a skin tunnelled catheter on an outpatient basis using a portable infusion pump. Treatment was continued for 26 weeks in the absence of dose-limiting toxicity (DLT, see below) or evidence of disease progression. Patients' measurable disease was reassessed at 12 weeks to assess tumour response. In this study, DLT was defined as: CTC grade III leucopenia (WBC ≤ 2.0 × $10^9$ $l^{-1}$), CTC grade II thrombocytopenia (platelets ≤ 75 × $10^9$ $l^{-1}$), *any* CTC grade II non-haematological toxicities (unless; nausea and vomiting controlled by anti-emetic therapy or diarrhoea controlled by loperamide). Any CTC grade III toxicity resulted in the immediate discontinuation of PVI 5FU. PVI 5FU could be recommenced following resolution of DLT, but the administered dose was reduced by 20%. Toxicity was assessed using standard CTC criteria where applicable. Hand–foot syndrome was classified using:

| | |
|---|---|
| Grade 0 | None |
| Grade 1 | Mild erythema, pain, dysaesthesias, and/or oedema; minimal fissures. Does not interfere with daily living |
| Grade 2 | Moderate erythema, pain, dysaesthesias, and/or oedema; moderate fissures. Interferes with daily living |
| Grade 3 | Severe erythema, pain, dysaesthesias, and/or oedema; Ulceration, necrosis or desquamation. Toxicity incapacitating |

### Blood sampling for 5FU pharmacokinetics

Plasma 5FU concentration was measured weekly to allow the assessment of both inter- and intra-subject variability. ALL samples were taken between 10.00 and 12.00 to minimize the variation due to circadian rhythm (Harris et al, 1990) and any samples recorded as being outwith the 10.00–12.00 time period were discarded. Blood samples were placed on ice immediately and spun within 15 minutes (Sorvall benchtop centrifuge, 3000 rpm for 5 minutes). The plasma fraction was then collected and frozen at –40˚C until the HPLC analysis was performed.

### HPLC analysis of 5FU

The HPLC assay used was a modification of that of Seymour et al 1994. 300 mg ammonium sulphate was added to 0.5 ml plasma, to precipitate plasma protein. 5 ml of a mixture of diethyl ether: propan-2-ol (80/20, %v/v) was added and the tubes were vortexed. The organic phase was back-extracted using 500 μl of 0.05M dipotassium hydrogen orthophosphate (pH 10.7). The solution was mixed and the supernatant discarded. 100 μl of the aqueous phase was transferred to a mini-Eppendorf and acidified with 20 μl of 1M orthophosphoric acid. 50 μl of this extract was injected onto the HPLC (Waters 2690 Alliance System, Waters 490E detector).

A 2 cm pre-column of Spherisorb Octadecyl Silane (ODS), 10 μm, was followed by a 15 cm analytical column of Apex ODS, 5 μm. 0.05 M potassium dihydrogen orthophosphate was used as the mobile phase with the pump set at a flow rate of 1.0 ml $min^{-1}$ and the column kept at 35˚C. Detection was at 270 nm, run time was 7 minutes, with a 5FU peak retention time of approximately 3.8 minutes. The limit of quantification was 20 ng $ml^{-1}$. Day-to-day interassay variability was 5% and intra-assay variability at 100 ng $ml^{-1}$ was 6%.

### Simulation of PK data for a similar population of patients

The model of 5FU pharmacokinetics described by Seymour et al (1994) was used to simulate 58 data sets for 'patients' receiving the PVI 5FU schedule at a dose of 300 mg $m^{-2}$. This model assumes a single compartment with linear and non-linear elimination processes, parameterised using Vd (mean = 10.4 ± 1.91 $m^{-2}$), Kel (mean = 0.0725 ± 0.0167 $min^{-1}$), Vmax (mean = 0.394 ± 0.126 mg $l^{-1}$ $min^{-1}$) and Km ('fixed' = 1.95 mg $l^{-1}$). The simulation was performed using ADAPT II (D'Argenio and Schumitsky).

### RESULTS

### Patients and characteristics

64 patients were entered into the study between July 1995 and July 1997. Of these, 3 were deemed ineligible on review of radiology (2 patients, no measurable disease) or subsequent pathology/clinical review (1 patient) which was more suggestive of a gastric primary tumour. All 61 eligible patients (male, 39; female 22) have completed treatment and the median follow up is 19.3 months. Patients were aged between 33 and 78 years (median 61 years). Performance status was good (PS 0, 49%; PS 1, 43%; PS 2, 8%) in most patients. Liver metastases were present in 51/61 patients (84%) and represented the only site of metastatic disease in 26 (43%).

PVI 5FU treatment duration varied from 1–26 weeks, with the median duration = 21 weeks and 24 patients (39%) achieving the full 26 week of intended therapy. Reasons given for discontinuation of treatment were disease progression, 26 (43%); toxicity, 9 (11%); completion of planned therapy, 24 (39%). There was 1 early death, attributed to 5FU cardiotoxicity, and 1 patient discontinued therapy due to worsening chronic obstructive airways disease. 20 patients (33%) required dose reduction for toxicity at some point during their treatment course.

### Response and survival data for the study population

In the 61 eligible patients, formal radiological assessment of response took place following 12–13 weeks of therapy and was only repeated at the end of planned treatment (26 weeks), unless clinically appropriate. Objective complete (1 patient, assessed by ultrasound only) or partial responses were recorded in 16/61 patients (26%, 95% CI 16–38%). There have been 52 deaths in the study population and the median survival for the population

© 2001 Cancer Research Campaign was 11 months. 26 of the 61 patients entered (43%) were alive at 1 year.

### Toxicity

The PVI 5FU regimen was generally well tolerated, although 47% of patients required delay and/or dose reduction due to toxicity. Dose reduction occurred on 31 occasions and these were attributed to hand–foot syndrome on 19 occasions (61%), diarrhoea on 5 occasions (16%) and stomatitis on 2 occasions (6%). The overall incidence of toxicities is shown in Table 1. Treatment was discontinued in 4 patients (7%) following Hickman line complications; thrombus (2), pain or accidental removal by the patient.

### Plasma pharmacokinetics of 5-FU

5FU pharmacokinetic data at the initial 5FU dose were available from 58 patients (3 patients discontinued treatment at 300 mg $m^{-2}$ day $^{-1}$ before reassessment; 1 due to early dose reduction, 1 due to rapid disease progression and 1 death due to myocardial ischaemia). The median number of samples/patient was 8 (range 1–23). The absolute range of concentrations was 30–260 ng $ml^{-1}$. The mean 5FU Css was calculated for each patient whilst receiving full dose (300 mg $m^{-2}$ $day^{-1}$) and used for comparative purposes. This value is referred to subsequently as '$5FU_{300}Css$'. The distribution of $5FU_{300}Css$ is shown in Figure 1.

Although there was some INTER-patient variability (mean; 94 ng $ml^{-1}$, SD 25, coefficient of variability (CV) = 27%), this was much less than had been predicted from the data of Findlay et al when the study was initiated. The degree of INTRA-patient variability was not excessive (CV; 5–39% (mean = 20%)) in the majority of patients. However, in 1 patient there was extensive course to course variability: 5FU Css range 88–260 ng $ml^{-1}$ (mean = 157, $n$ = 10).

The results from the simulation performed were similar to those measured: simulation mean = 88 ng $ml^{-1}$ (range 45–240 ng $ml^{-1}$); measured 94 ng $ml^{-1}$ (range 30–260 ng $ml^{-1}$).

### Relationships of 5FU concentration, patient characteristics and outcome.

Outcome measures such as response and toxicity were compared to $5FU_{300}Css$. This allows comparison between patients as it excludes the impact of duration of therapy and dose reductions on correlative analyses. Survival was analysed by 5FU concentration, divided into quartiles, but this did not show any statistically significant advantage for higher 5FU concentrations.

Results (selected) of these analyses are shown in Table 2. In addition, it was also noted that stomatitis occurred more commonly ($P < 0.003$) in women. Previously, it has been reported that DPD activity is 15% lower in women (Etienne et al, 1994) and this might lead to a gender difference in plasma 5FU concentrations, as described by Vokes et al (1996). Also, in the Vokes study, higher 5FU concentrations were associated with an increase in mucositis. However, in our study mucositis was not related to 5FU concentration and there was no difference between males and females in relation to plasma concentrations ($P = 0.5156$). Indeed, other authors (Lu et al, 1993) have reported *higher* DPD enzyme activity in women. Clearly more work is required in this area. In addition, in our study, males appeared more likely to respond, but this is difficult to explain and has not been reported previously. It is possible that these apparent relationships may represent artefacts of multiple testing.

Analyses were also performed using total exposure to 5FU over time, but this did not provide additional information compared to the data for $5FU_{300}Css$, shown in Table 2.

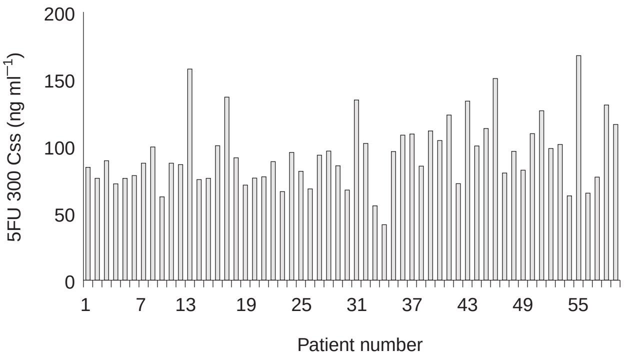

**Figure 1** Mean $5FU_{300}$ Css per patient

### DISCUSSION

PVI 5FU activity in patients with metastatic colorectal cancer has been confirmed. The overall response rate was 26% and the median survival was 11 months. The response rate in the original phase III trial of this regimen was 30% (Lokich et al, 1988) and in the recently published overview, the response rate for PVI 5FU was 22% (Meta-Analysis Group in Cancer, 1998). PVI 5FU was generally well tolerated, although more than half the patients (52%) required at least 1 break during the 26 weeks of therapy and 20 patients (33%) required a dosage reduction.

The pharmacokinetic data presented are consistent with previously published data. In the simulation presented, a population mean $5FU_{300}Css$ of 88 ng $ml^{-1}$ was predicted (94 ng $ml^{-1}$ in our data set) and the predicted range of concentrations, 45–240 ng $ml^{-1}$ (compared to 30–260 ng $ml^{-1}$). This comparison also demonstrates the applicability of the Seymour model to patient data generated using an alternative method of 5FU administration. In contrast, in an early paper, Harris et al reported lower 5FU Css

**Table 1**

| Toxicity | Percentage of patients at each CTC grade ($n$ = 61) | | | | |
|---|---|---|---|---|---|
| | 0 | 1 | 2 | 3 | 4 |
| Stomatitis | 35 | 42 | 18 | 5 | 0 |
| Hand/foot | 20 | 43 | 22 | 15 | N/A |
| Diarrhoea | 32 | 32 | 25 | 12 | 0 |
| Hickman complications | 22 | 33 | 40 | 5 | N/A |

**Table 2**

| Independent variable | Dependent variable | $P$ value |
|---|---|---|
| $5FU_{300}Css$ | Diarrhoea | 0.164 |
| $5FU_{300}Css$ | Hand–foot | 0.410 |
| $5FU_{300}Css$ | Stomatitis | 0.949 |
| $5FU_{300}Css$ | Dose reduction | 0.941 |
| $5FU_{300}Css$ | Tumour response | 0.182 |

© 2001 Cancer Research Campaign values (27.4 ± 1.3 ng $ml^{-1}$) at 11 a.m. in patients receiving PVI 5FU at this dose. However, only 7 patients were included in that study (Harris et al, 1990).

The inter-subject variability in $5FU_{300}Css$ (CV = 34%) in this study is, considerably less than that reported by Findlay et al (1996). The data of Findlay et al require some discussion as the range of concentrations they reported was particularly large. Indeed, the higher concentrations were higher than those reported for much more intense 5-FU administration protocols (Gamelin et al, 1998). Questions could be asked about the PK sampling and subsequent sample handling, although the authors were careful to state that plasma sampling was not from the Hickman line explaining the high concentrations and that there was no apparent delay in freezing and spinning samples to explain the low concentrations. Etienne et al (1995) have drawn attention to the mechanism of action of pump devices as most deliver multiple small boluses and the temporal relationship between these dosing ‘surges’ and sampling, may add imprecision to PK data. However, intrapatient variability, which might in part be explained by fluctuations in the rate of drug administration, was relatively small in our study.

In summary, the activity of PVI 5FU in patients with metastatic colorectal cancer was confirmed (RR = 26%) and the regimen was generally well tolerated. Intrasubject variability in $5FU_{300}Css$ was not excessive (mean CV = 20%) and was less than inter-subject variability in $5FU_{300}Css$ (CV = 27%). Inter-subject variability was considerably less than that reported by Findlay et al. The lack of any correlation between $5FU_{300}Css$ and measures of outcome suggest that measurement of 5FU concentrations should not be used to individualise dosing in patients receiving PVI 5FU for advanced colorectal cancer.

### REFERENCES

D’Argenio DZ and Schumitsky A. ADAPT II User’s Guide, Biomedical Simulations Resource, University of Southern California, Los Angeles, CA

Etienne MC, Milano G, Lagrange JL, Bajard F, Francois E, Thyss A, Schneider M, Renee N and Fety R (1993) Marked fluctuations in drug plasma concentrations caused by use of portable pumps for fluorouracil continuous infusion (letter). *J Natl Cancer Inst* **85**(12): 1005–7

Etienne MC, Lagrange JL, Dassonville O, Fleming R, Thyss A, Renee N, Schneider M, Demard F and Milano G (1994) Population study of dihydropyrimidine dehydrogenase in cancer patients. *J Clin Oncol* **12**: 2239–2242

Fety R, Rolland F, Campion L, Perrocheau G, Merlin JL, Barberi-Heyob M, Conroy T, Hardouin A, Riviere A and Milano G (1997) A multicentric randomised trial of 5-fluorouracil (FU) dose adaptation (DA) based on pharmcokinetics (PK). Clinical and Economic Impacts. *Proc Am Soc Clin Oncol* **16**: 225a

Findlay MPN, Raynaud F, Cunningham D, Iveson A, Collins DJ and Leach MO (1996) Measurement of plasma 5FU by HPLC with comparison of results to tissue drug levels observed using in vivo MRS in patients on a PVI with or without interferon a. *Annal Oncol* **7**: 47–53

Fleming RA, Milano G, Thyss A, Etienne MC, Renee N, Schneider M and Demard F (1992) Correlation between dihydropyrimidine dehydrogenase-activity in peripheral mononuclear cells and systemic clearance of fluorouracil in cancer patients. *Cancer Res* **52**(10): 2899–2902

Gamelin E, Bosidron-Celle M, Delva R, Regimbeau C, Cailleux PE, Alleaume C, Maillet ML, Goudier MJ, Sire M, Person-Joly MC, Maigre M, Maillart P, Fety R, Burtin P, Lortholary A, Dumesnil Y, Picon L, Geslin J, Gesta P, Danquechin-Dorval E, Larra F and Robert J (1998) Long-term weekly treatment of colorectal metastatic cancer with fluorouracil and leucovorin: results of a multicentric prospective trial of fluorouracil dosage optimisation by pharmacokinetic monitoring in 152 patients. *J Clin Oncol* **16**(4): 1470–1478

Harris BE, Carpenter JT and Diasio RB (1991) Severe 5-fluorouracil toxicity secondary to dihydropyrimidine dehydrogenase deficiency. A potentially more common pharmacogenetic syndrome. *Cancer* **68**(3): 499–501

Harris BE, Song R, Soong S and Diasio RB (1990) Relationship between dihydropyrimidine dehydrogenase activity and plasma 5-fluorouracil levels with evidence for circadian variation of enzyme activity and plasma drug levels in cancer patients receiving 5-fluorouracil by protracted continuous infusion. *Cancer Res* **50**: 197–201

Lokich JL, Ahlgren JD, Gullo JJ, Philips JA and Fryer JG (1988) A prospective randomised comparison of continuous infusion fluorouracil with a conventional bolus schedule in metastatic colorectal carcinoma: a mid-Atlantic oncology program study. *J Clin Oncol* **7**: 425–432

Lu Z, Zhang R and Diasio RB (1993) Dihydropyrimidine dehydrogenase activity in human peripheral blood mononuclear cells and liver: population characteristics, newly identified deficient patients and clinical implication in 5-fluorouracil chemotherapy. *Cancer Res* **53**(22): 5433–5438

Meta-analysis Group in Cancer (1998) Efficacy of intravenous continuous infusion of fluorouracil compared with bolus administration in advanced colorectal cancer. *J Clin Oncol* **16**: 301–308

Santini J, Milano G, Thyss A, Renee N, Viens P, Ayela P, Schneider M and Demard F (1989) 5FU therapeutic monitoring with dose adjustment leads to an improved therapeutic index in head and neck cancer. *Br J Cancer* **59**(2): 287–90

Seymour MT, Patel N, Johnston A, Joel SP and Slevin ML (1994) Lack of effect of interferon 2α upon fluorouracil pharmacokinetics. *Br J Cancer* **70**: 724–728

McMurrough and McLeod (1996) Analysis of the DPD polymorphism in a British population. *Br J Clin Pharmacol* **41**: 425–427

Vokes BE, Mick R, Kios MS, Dolan ME, Malone D, Athanasiadis J, Daraf DJ, Kozloff M, Weichselbaum RR and Ratain MJ (1996) Pharmacodynamics of fluorouracil-based induction chemotherapy in advanced head and neck cancer. *J Cancer Oncol* **14**(5): 1663–71

*© 2001 Cancer Research Campaign*
